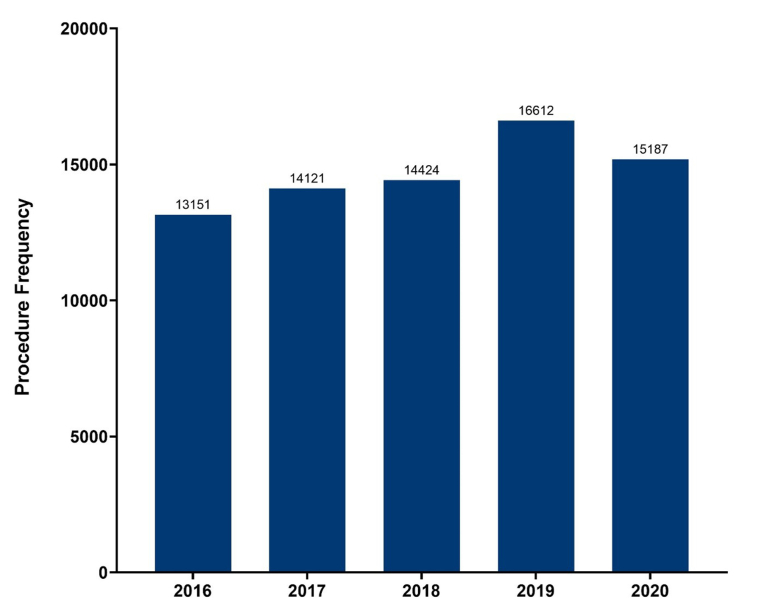# National Trends in Mitral Valve Surgery Outcomes in Centers With and Without Mitral Transcatheter Edge-to-Edge Repair

**DOI:** 10.1016/j.atssr.2025.06.012

**Published:** 2025-07-17

**Authors:** Sophia R. Pyeatte, Maxwell C. Braasch, Cameran Jones, Theodore Marghitu, Kyle Stumbaugh, June He, Alexander A. Brescia, Harold G. Roberts, Nicholas Kouchoukos, Tsuyoshi Kaneko

**Affiliations:** 1 Division of Cardiothoracic Surgery, Department of Surgery, Washington University in St. Louis, St. Louis, Missouri

## Abstract

**Background:**

Valve guidelines recommend mitral valve transcatheter edge-to-edge repair (M-TEER) be offered at comprehensive valve centers along with complex mitral valve surgery (MVS). We evaluated mitral valve surgery (MVS) outcomes based on center availability of M-TEER on a national scale.

**Methods:**

The National Readmissions Database was used to review adult patients who underwent MVS at centers with and without M-TEER from 2016 to 2020. Patients with a history of endocarditis or prosthetic valve dysfunction were excluded. The primary outcome was 30-day mortality. Multivariable logistic regression analysis was conducted to determine the effect of M-TEER availability on postoperative mortality.

**Results:**

Of the 50,179 patients who underwent MVS from 2016 to 2020, 15,485 underwent MVS at a non–M-TEER hospital. During this period, the number of centers with M-TEER significantly increased from 2539 in 2016 to 6326 in 2020 (*P* < .05). The annual volume of M-TEER procedures performed significantly increased, whereas there was no significant change in the annual volume of MVS. Patients at non–M-TEER hospitals tended to be older, male, with higher rates of comorbidities and prior cardiac interventions (all *P* < .05). MVS 30-day mortality was significantly higher at non–M-TEER centers than at M-TEER centers (6.7% vs 5.0%, *P* < .001). Multivariable analysis showed non–M-TEER hospital status was independently associated with higher 30-day mortality (odds ratio 1.21; 95% CI 1.09-1.33) after MVS.

**Conclusions:**

Centers with M-TEER have significantly lower 30-day mortality after MVS than centers without M-TEER. This study supports the concept of a comprehensive valve center in the treatment of mitral valve disease.

## Visual Abstract

**Figure:**
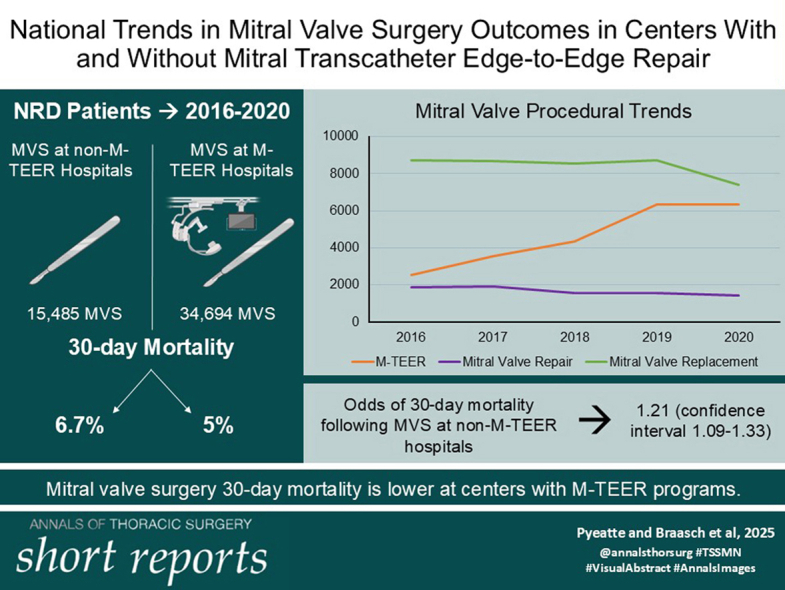

**In Short**

▪ Centers with mitral valve transcatheter edge-to-edge repair have significantly lower 30-day mortality after mitral valve surgery than centers without mitral valve transcatheter edge-to-edge repair.
▪ The concept of a comprehensive valve center should be pursued in the treatment of mitral valve disease.

Mitral regurgitation (MR) is the most common valvular disease in the United States. Treatment of primary MR was revolutionized in 2013 with mitral valve transcatheter edge-to-edge repair (M-TEER) for primary MR^1^ and subsequent evaluation in patients with secondary MR.^2^ M-TEER use has expanded rapidly and now has class IIA recommendations for patients with severe symptomatic primary MR or prohibitive surgical risk as well as patients with secondary MR.^3^ Initiation of M-TEER programs is dependent on each center’s mitral valve surgery (MVS) volume, based on the National Coverage Determinations issued by the Centers for Medicare and Medicaid Services, which also includes cardiac surgeon and interventional cardiologist annual procedural volume requirements.^4^

The 2020 Valve Guidelines suggested the valve centers of excellence concept with level I comprehensive valve centers performing M-TEER and complex MVS.^3^ Although the introduction of M-TEER is associated with improved mortality in patients undergoing mitral valve repair (MVr) at the institutional level,^5^ the national impact of M-TEER availability on MVS outcomes is unknown. In this study, we examined outcomes of MVS based on center availability of M-TEER on a national scale. We hypothesized that centers with M-TEER would have better outcomes after MVS due to use of a multidisciplinary team approach and the triaging of higher-risk patients to M-TEER.

## Patients and Methods

### Data Source

This study used the Nationwide Readmissions Database (NRD), a large national database that includes nationwide all-payer inpatient care. International Classification of Disease diagnosis and procedure codes were used for data collection. The Washington University in St. Louis Institutional Review Board determined this study to be exempt, and informed consent was waived (IRB #202404142; approved April 23, 2024).

### Study Design and Population

**Supplementary Figure 1:**
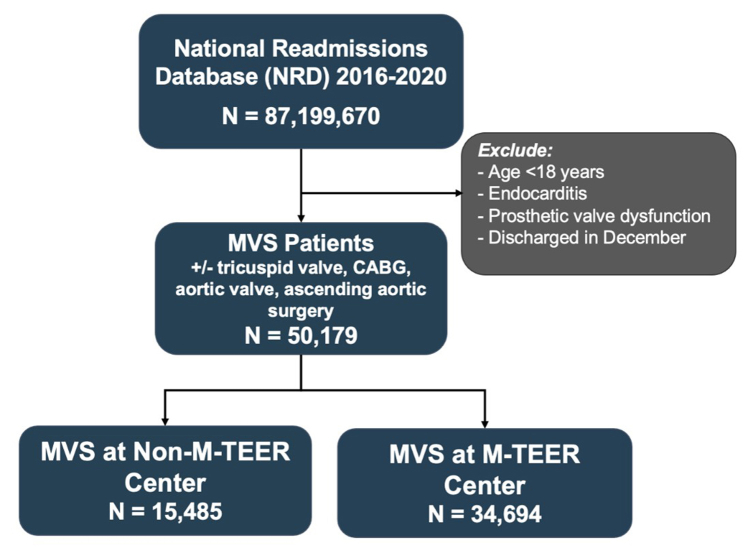


Patients aged ≥18 years who underwent MVS at centers with and without M-TEER from 2016 to 2020 were included (Supplemental Figure 1). Patients with a history of endocarditis or prosthetic valve dysfunction were excluded. Patients were grouped by the presence of M-TEER at their center.

### Variables and Outcomes of Interest

The primary outcome was 30-day mortality after MVS. Secondary outcomes included annual MVS volume, 30-day readmission, and postoperative complications.

### Statistical Analysis

Continuous variables are reported as mean (SD) and were compared using *t* tests. Categorical variables were compared with χ^2^ analysis. Annual center MVS volume was stratified into tertiles to classify centers as low, intermediate, or high volume. Multivariable logistic regression analysis was conducted to determine the effects of TEER availability on 30-day mortality. Sensitivity analyses were performed evaluating 30-day mortality for MVr only and mitral valve replacement (MVR) only. All analyses were performed using SAS 9.4 (SAS Institute Inc) statistical software.

## Results

### Procedural, Patient, and Center Characteristics

**Figure 1:**
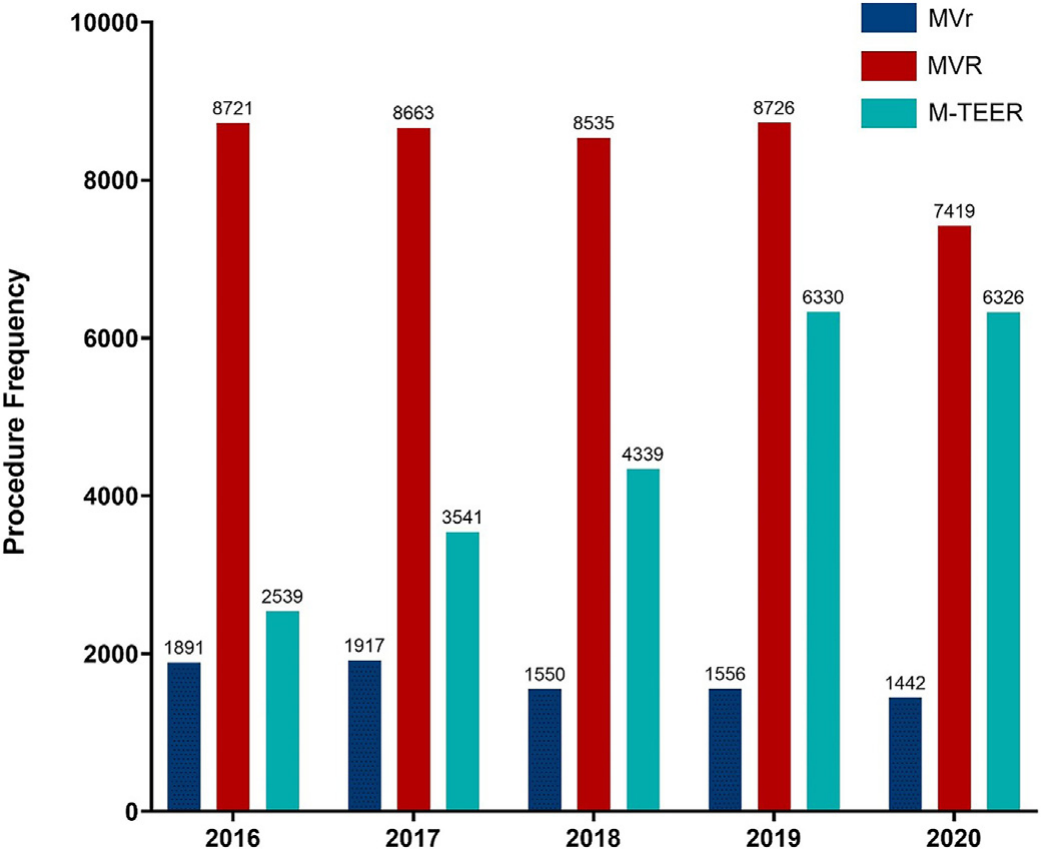
Annual mitral valve surgery and mitral transcatheter edge-to-edge repair (M-TEER) volume. (MVr, mitral valve repair; MVR, mitral valve replacement.)

There were 23,075 M-TEER procedures performed over the study period, increasing from 2539 in 2016 to 6326 in 2020 (Figure 1). Total mitral valve annual procedural volume increased from 2016 to 2019 (Supplemental Figure 2). Of the 50,179 patients who underwent MVS from 2016 to 2020, 15,485 underwent MVS at a non–M-TEER hospital and 34,694 underwent MVS at an M-TEER hospital (Table). Patients at non–M-TEER hospitals were older with greater comorbidity burden (all *P* < .05). The number of centers with M-TEER significantly increased from 2016 to 2020 (*P* < .05).

**Table:** Patient Characteristics and Periprocedural Outcomes

Variable	MVS at Non–M-TEER Hospitals	MVS at M-TEER Hospitals	*P* Value
	(n = 15,485)	(n = 34,694)	
Age, mean (SD), y	66 (12)	65 (12)	<.001
Female sex	7852 (50.7)	18,023 (51.9)	.01
Hypertension	4772 (30.8)	9037 (26.1)	<.001
Dyslipidemia	8378 (54.1)	18,950 (54.6)	.29
Diabetes	4082 (26.4)	8965 (25.8)	.22
Peripheral vascular disease	706 (4.6)	1396 (4.0)	.006
COPD	2597 (16.8)	4884 (14.1)	<.001
Cerebrovascular disease	86 (0.6)	166 (0.5)	.29
Kidney disease	3476 (22.5)	8118 (23.4)	.02
Coronary artery disease	403 (2.6)	1446 (4.2)	<.001
Prior myocardial infarction	1313 (8.5)	2927 (8.4)	.89
Congestive heart failure	718 (4.6)	1327 (3.8)	<.001
Prior PCI	158 (1.0)	287 (0.8)	.04
Atrial fibrillation	4404 (28.4)	10,094 (29.1)	.14
Previous CABG	758 (4.9)	1570 (4.5)	.07
Periprocedural outcomes			
Mitral valve repair	2395 (15.5)	5961 (17.2)	<.001
30-day mortality	1033 (6.7)	1715 (5.0)	<.001
30-day readmission	856 (6.0)	1880 (5.7)	.37
Stroke	9 (0.0006)	15 (0.0004)	.60
Renal failure	3912 (27.2)	9046 (27.6)	.37
Cardiac arrest	200 (1.4)	409 (1.2)	.23
Permanent pacemaker	1586 (11.0)	3419 (10.4)	.06
Complete heart block	2036 (14.2)	5726 (17.5)	<.001
Bleeding	446 (3.1)	1032 (3.1)	.80
Ventricular arrhythmia	37 (0.2)	97 (0.3)	.47
Pneumonia	1453 (10.1)	3179 (9.7)	.19

Values are displayed as frequencies (%) or as mean (SD), as indicated.

CABG, coronary artery bypass graft; COPD, chronic obstructive pulmonary disease; MVS, mitral valve surgery; M-TEER, mitral valve transcatheter edge-to-edge repair; PCI, percutaneous coronary intervention.

### Mitral Valve Surgery Outcomes

**Figure 2:**
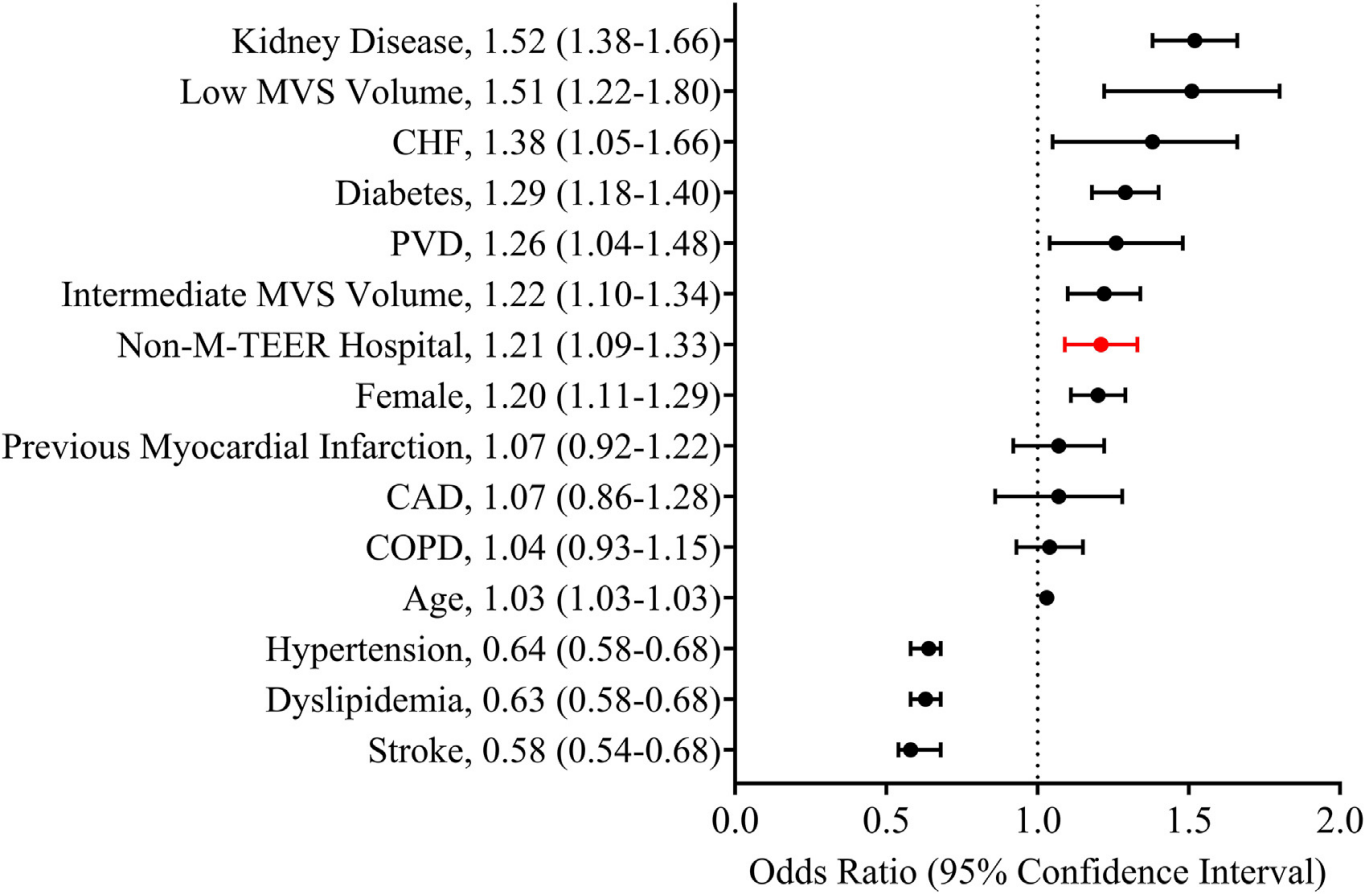
Multivariable analysis of 30-day mortality after mitral valve surgery (MVS). (CAD, coronary artery disease; CHF, congestive heart failure; COPD, chronic obstructive pulmonary disease; CVD, cerebrovascular disease; M-TEER, mitral valve transcatheter edge-to-edge repair; PVD, peripheral vascular disease.)

Mortality at 30 days after MVS was significantly higher at non–M-TEER centers than at M-TEER centers (6.7% vs 5.0%, *P* < .001) (Table). The percentage of MVS performed as MVr was lower at non–M-TEER centers than at M-TEER centers (15.5% vs 17.2%, *P* < .001). Patients at non–M-TEER centers had similar 30-day readmission rates after MVS compared with patients at M-TEER centers (6.0% vs 5.7%, *P* = .37). Multivariable analysis showed non–M-TEER hospital status was independently associated with higher 30-day mortality after MVS (odds ratio [OR] 1.21; 95% CI 1.09-1.33) (Figure 2).

### Sensitivity Analysis: MV Repair and Replacement

**Figure 3:**
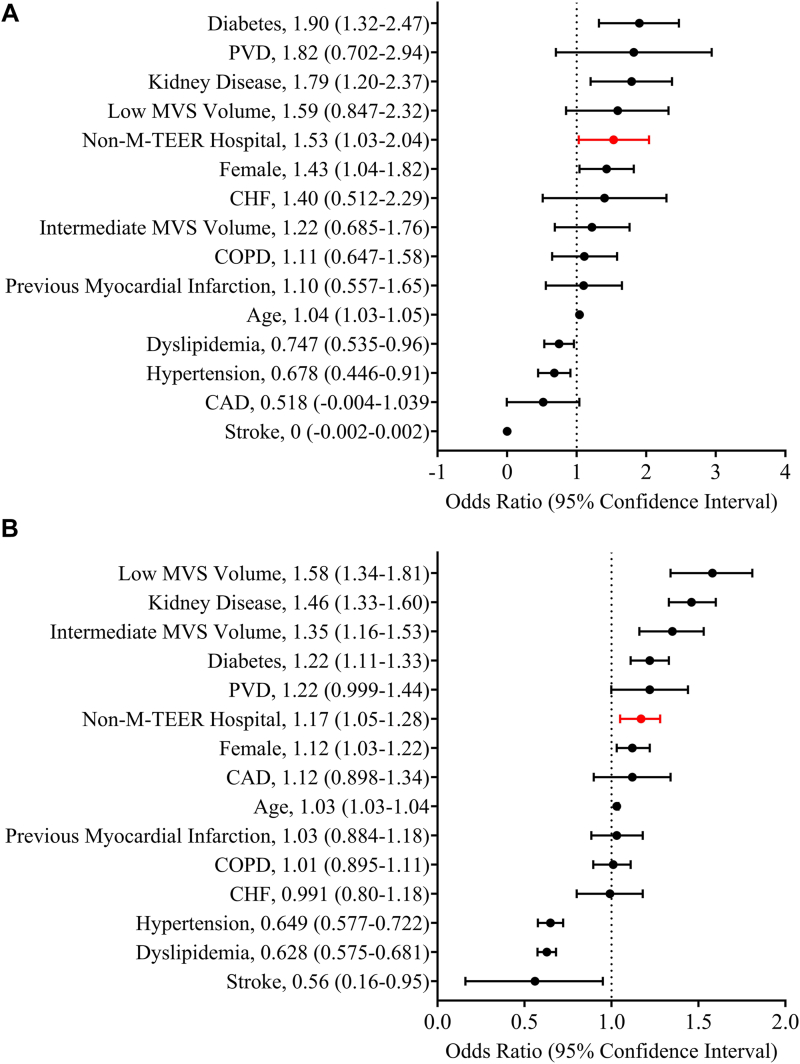
Multivariable analysis of 30-day mortality after (A) mitral valve repair and (B) mitral valve replacement. (CAD, coronary artery disease; CHF, congestive heart failure; COPD, chronic obstructive pulmonary disease; CVD, cerebrovascular disease; MVS, mitral valve surgery; M-TEER, mitral valve transcatheter edge-to-edge repair; PVD, peripheral vascular disease.)

On multivariate analysis for MVr only, non–M-TEER center status was associated with higher 30-day mortality (OR 1.53; 95% CI 1.03-2.04) (Figure 3). On multivariate analysis for MVR only, non–M-TEER center status was associated with higher 30-day mortality (OR 1.17; 95% CI 1.05-1.28) (Figure 3).

## Comment

This nationwide analysis of MVS outcomes by M-TEER center status reports several significant findings. The annual volume of M-TEER is increasing, with no significant change in annual MVS volume. M-TEER centers have significantly lower 30-day mortality after MVS than non–M-TEER centers. Non–M-TEER center status is independently associated with higher 30-day mortality after MVS, MVr, and MVR. These findings suggest that level I comprehensive valve centers that offer M-TEER may allow higher-risk patients to be treated with surgical and transcatheter interventions with satisfactory outcomes, supporting the concept of centralizing higher-risk cases to these centers.

The annual volume of M-TEER is increasing, without a significant change in annual MVS volume. Analysis of the Society of Thoracic Surgeons–American College of Cardiology Transcatheter Valve Therapy registry from 2014 through early 2020 demonstrated a similar increase in M-TEER each year.^6^ Lowenstern and colleagues^5^ also reported no difference in annual MVr volume in a multi-institutional study examining institutional MVr outcomes before and after introduction of M-TEER.^5^ By contrast, Chikwe and colleagues^7^ reported a 30.7% decrease in MVr between 2012 and 2019 with a corresponding 23-fold increase in M-TEER volume. However, this decrease in MVr plateaued in 2016, with MVr volume relatively stable by 2019.^7^ Short-term mortality after M-TEER may not be affected by center MVr volume; however, intermediate-term mortality is lower at high-volume MVr centers, suggesting a complex relationship yet to be fully understood.^8^

We hypothesize that improved mortality after MVS at M-TEER centers may be secondary to patients with higher surgical risk undergoing M-TEER as opposed to MVS, not that surgical therapy is necessarily superior at M-TEER centers. Lower-risk surgical patients are being directed towards MVS rather than M-TEER.^5^^,^^7^ As M-TEER expanded, 30-day mortality after MVr decreased from 2.4% in 2012 to 0.9% in 2019.^7^

Our observed 30-day mortality of 6.7% and 5.0% after MVS in non–M-TEER and M-TEER centers, respectively, could be attributed in part to a high proportion of patients with secondary MR. Although primary MR results from valvular degeneration, secondary MR, also known as function MR, is secondary to ventricular or atrial remodeling^9^ that hinders the function of a structurally normal valve. A recent analysis of The Society of Thoracic Surgeons data of patients with primary MR undergoing MVS reported operative mortality of ∼1.3%.^5^ Patients with secondary MR have poorer outcomes after a valvular intervention compared with patients with primary MR.^10^ Our study also included patients who underwent concomitant coronary artery bypass grafting, non-mitral valve surgery, and ascending aortic surgery, which increases operative risk.

To be designated as an M-TEER center, hospitals must have cardiologists and cardiac surgeons who meet certain procedural requirements.^4^ The rational for the hospital volume for M-TEER is based on the resource support needed for the procedure. Moreover, the multidisciplinary heart team is critical in deciding the strategy for complex patients. Our findings should be interpreted as support for the concept of the center for excellence^3^ for comprehensive care for valvular heart disease in which higher-risk patients have access to more appropriate procedures and more experienced, higher-volume care teams. Mitral centers of excellence should continue to offer a plethora of surgical and transcatheter interventions, multidisciplinary expertise, and extensive facility capabilities as outlined by the American College of Cardiology, the American Heart Association, and the Mitral Foundation. This diverse and knowledgeable team offering a greater variety of mitral interventions may play a significant role in improved MVS outcomes at M-TEER centers.

### Limitations

Our study has limitations. This study is reliant on administrative claims data, which do not provide detailed imaging or laboratory data. International Classification of Diseases, 10th Edition, coding is subject to misclassification. It is not possible to track individual center outcomes before and after the introduction of M-TEER with NRD data. The NRD does not differentiate between primary and secondary MR. Selection bias cannot be accounted for in patient selection for MVS or M-TEER. Lastly, NRD data do not include data to calculate The Society of Thoracic Surgeons predicted risk of mortality, thereby limiting operative risk assessment.

### Conclusion

This analysis from the NRD demonstrates that centers with M-TEER have significantly lower 30-day mortality after MVS than centers without M-TEER, even after adjustment by MVS volume. This study supports the concept of centers of excellence in the treatment of mitral valve disease.

## Figures and Tables

**Supplementary Figure 2:**