# Supplementary material for: National Trends in Mitral Valve Surgery Outcomes in Centers With and Without Mitral Transcatheter Edge-to-Edge Repair
Source: Ann Thorac Surg Short Rep. 2025 Jul 17;3(4):1023–8. doi: 10.1016/j.atssr.2025.06.012 (PMC12712196; doi:10.1016/j.atssr.2025.06.012)
Supplement: Supplementary Figures 1 and 2 [file mmc1.docx]

**Supplemental Figure 1.** Study Design.

Abbreviations: CABG: coronary artery bypass graft; MVS: mitral valve surgery; M-TEER: mitral valve transcatheter edge-to-edge repair; NRD: National Readmissions Database.

**Supplemental Figure 2.** Annual Volume of M-TEER Plus Mitral Valve Surgery.
